# Differences in the Inhibitory Specificity Distinguish the Efficacy of Plant Protease Inhibitors on Mouse Fibrosarcoma

**DOI:** 10.3390/plants10030602

**Published:** 2021-03-23

**Authors:** Sonia Yoo Im, Camila Ramalho Bonturi, Adriana Miti Nakahata, Clóvis Ryuichi Nakaie, Arnildo Pott, Vali Joana Pott, Maria Luiza Vilela Oliva

**Affiliations:** 1 Departamento de Bioquímica, Universidade Federal de São Paulo (UNIFESP), 04044-020 São Paulo, SP, Brazil; yoo.sonia@gmail.com (S.Y.I.); camilabntr@gmail.com (C.R.B.); 2 Laboratório de Genômica e Biologia Molecular, Centro Internacional de Pesquisa (CIPE) do A.C.Camargo Cancer Center, 01508-010 São Paulo, SP, Brazil; adriana_miti@yahoo.com.br; 3 Departamento de Biofísica, Universidade Federal de São Paulo (UNIFESP), 04044-020 São Paulo, SP, Brazil; cnakaie@unifesp.br; 4 Departamento de Biologia, Universidade Federal de Mato Grosso do Sul (UFMS), Cidade Universitária, 79070-900 Campo Grande, MS, Brazil; arnildo.pott@gmail.com (A.P.); vali.pott@gmail.com (V.J.P.)

**Keywords:** fibrosarcoma, metastasis, natural products, plants, protease inhibitors, tumor cells

## Abstract

Metastasis, the primary cause of death from malignant tumors, is facilitated by multiple protease-mediated processes. Thus, effort has been invested in the development of protease inhibitors to prevent metastasis. Here, we investigated the effects of protease inhibitors including the recombinant inhibitors rBbKI (serine protease inhibitor) and rBbCI (serine and cysteine inhibitor) derived from native inhibitors identified in *Bauhinia bauhinioides* seeds, and EcTI (serine and metalloprotease inhibitor) isolated from the seeds of *Enterolobium contortisiliquum* on the mouse fibrosarcoma model (lineage L929). rBbKI inhibited 80% of cell viability of L929 cells after 48 h, while EcTI showed similar efficacy after 72 h. Both inhibitors acted in a dose and time-dependent manner. Conversely, rBbCI did not significantly affect the viability of L929 cells. Confocal microscopy revealed the binding of rBbKI and EcTI to the L929 cell surface. rBbKI inhibited approximately 63% of L929 adhesion to fibronectin, in contrast with EcTI and rBbCI, which did not significantly interfere with adhesion. None of the inhibitors interfered with the L929 cell cycle phases. The synthetic peptide RPGLPVRFESPL-NH_2_, based on the BbKI reactive site, inhibited 45% of the cellular viability of L929, becoming a promising protease inhibitor due to its ease of synthesis.

## 1. Introduction

Sarcomas are a heterogeneous group of malignant neoplasms that originate from mesenchymal cells and mostly affect soft tissues [1,2]. Fibrosarcoma is a rare malignant neoplasm that develops in connective tissue from fibroblasts and occurs in adults and children. The appearance of fibrosarcoma is more frequent in adults between the ages of 20 and 50, while its incidence is rare in children [3]. Fibrosarcoma is generally more aggressive in adults than in infants [1,3]. In general, fibrosarcoma presents as a palpable, painless mass (77% of cases), impairing early diagnosis. Further, fibrosarcoma is often falsely diagnosed as a traumatic hematoma or simple muscle strain due to a large number of existing histopathological subtypes [1,2]. The standard treatment for sarcomas includes surgical removal combined with radiation therapy. However, the protocol for marginal surgical resection, although of great importance, still has no medical consensus. The same is true for radiotherapy, in which a defined protocol is lacking for the duration or time of exposure, either before or after surgery [4,5]. Although sarcomas are rare tumors, the advanced stage is difficult to treat, with a survival rate below 57% and a higher incidence of recurrence [1,6]. Adjuvant therapies for surgery and radiotherapy, such as chemotherapy and target therapy, are also needed, mainly in high-grade fibrosarcoma [1]. Chemotherapeutic drugs are often combined with target therapy or immunotherapy to increase the response of unresectable or metastatic sarcomas [6,7]. However, surgery can result in limb amputation, causing physical and mental damage to patients. In addition to high recurrence rates of fibrosarcoma, available treatments have serious side effects, such as heart failure and severe or fatal hepatotoxicity.

Proteases play a fundamental role in numerous biological processes, including cell cycle progression, replication of genetic material, the immune response, cell adhesion, proliferation, migration, acting in signaling biomolecules, and apoptotic processes, all of which interfere with tumor progression. These numerous functions make them essential targets in the study of various pathophysiological processes, such as cancer [8]. In fibrosarcoma, a variety of proteases have been related to tumor progression, metastasis, and response to chemotherapy [8,9]. In this view, protease inhibitors are instruments used for the study and development of new therapeutic strategies.

Many protease inhibitors have been isolated from plants, mainly from legume seeds [10,11,12,13,14]. One aspect of interest is to investigate the relationship between the structure, specificity, and selectivity of different proteases and their enzymatic activity, which is an important factor in the development of drugs such as enzymes that affect the processes of cancer progression [8,9,15].

Studies with protease inhibitors have demonstrated that their cytostatic/cytotoxic activity may be the result of direct inhibition of matrix proteolysis or indirect inhibition of proteolytic cascade activation, thus preventing the spread of tumor cells [8]. Their effectiveness has been demonstrated in various studies, including in breast cancer cell lines [15] and the control of asthma in mice [16]. In particular, inhibitors derived from *Bauhinia bauhinioides*, BbKI (human plasma kallikrein inhibitor) and BbCI (cruzipain inhibitor), were investigated in models of inflammation and reperfusion [10,17], venous and arterial thrombosis of mice [13], a prostate cancer model [18], cell invasion and angiogenesis in different cell lines [12], a pulmonary emphysema model in mice [14], and as a potential insecticidal agent [19]. EcTl, a trypsin inhibitor derived from *Enterolobium contortisiliquum*, was investigated in gastric cancer [11], triple-negative breast cancer [20], glioblastoma [21,22], the reduction of inflammation and pulmonary remodeling in induced lung inflammation [23], and in an asthma model [24]. In addition, EcTI was investigated in non-tumorigenic cells such as human mesenchymal cells [25] and showed no effects, demonstrating the potential use of these compounds as therapeutic targets. Considering that in fibrosarcoma the tumor establishment is mediated by multiple actions of proteases that cooperate with their differences in specific action [10,26], an aspect of interest in this study was to investigate the effects of plants inhibitors, with different specificities, that may help to understand the proliferative activity of fibrosarcoma. Thus, here we demonstrated the effects of EcTI and the recombinant inhibitors rBbCI and rBbKI on mouse fibrosarcoma cells.

## 2. Results

### 2.1. Inhibitor Purification and Characterization

Native and recombinant inhibitors were obtained in sufficient amounts for the experiments. Inhibitor purity was confirmed by electrophoresis and reverse-phase chromatography as published previously by Martins-Oliveira et al. [14], de Paula et al. [11], and Almeida-Reis et al. [27], and their inhibitory properties are shown in Table 1. Inhibitory activity of recombinant and native forms of the protein BbCI were maintained, as cruzain, or even improved, as human neutrophil elastase (HNE) and cruzipain. HNE activity has been upregulated in many cancers and frequently correlated to poor clinical outcomes [28]. Recombinant BbKI inhibited trypsin, human plasma kallikrein (PKa), and porcine pancreatic kallikrein, as the native protein. PKa inhibition has been related to cell growth, angiogenesis, invasion, and metastasis of a variety of cancers [29].

The recombinant forms of BbKI and BbCI facilitated obtaining these inhibitors in high amounts since *B. bauhinioides* grows in a region of Brazil with limited access, whereas *E. contortisiliquum* is widely distributed throughout Brazil. EcTI and BbKI are both serine protease inhibitors that contain arginine at the P1 position of the reactive site [10,26]; however, specificity differences demonstrated by these proteins made them interesting candidates for cellular studies, especially those of tumor origin.

### 2.2. Effects of rBbCI, rBbKI, and EcTI on the L929 Fibrosarcoma Lineage

#### 2.2.1. Effects of Inhibitors on Cell Viability

We investigated the effects of these inhibitors on fibrosarcoma cells (L929) since they have not yet been evaluated in malignant sarcoma cell lines and may be important tools in the study to understand the cellular processes involved in this type of tumor.

**Figure 1:**
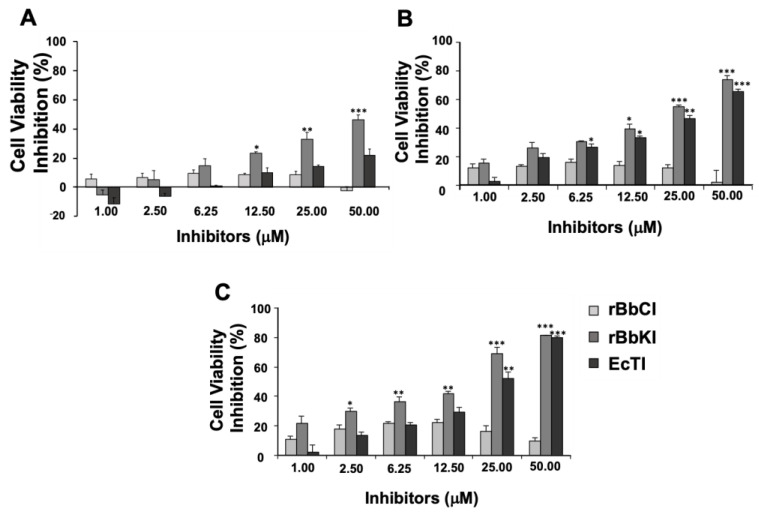
Action of rBbCI, rBbKI, and EcTI on the cell viability of L929 cells. L929 cells were pre-incubated for 24 h, increasing concentrations of rBbCI, rBbKI, and EcTI were added to the 96-well plates, and cells were analyzed after incubation for (**A**) 24 h, (**B**) 48 h, (**C**), or 72 h. Each bar represents the mean ± standard deviations of three repetitions. (* *p* <0.05, ** *p* <0.005, *** *p* <0.0001; One way-ANOVA, follow Tukey’s Multiple Comparison Test).

The effects of rBbCI, rBbKI, and EcTI (1.0–50 µM) on cell viability were evaluated at different incubation times (24, 48, and 72 h). rBbKI inhibited L929 cell viability in a dose and time-dependent manner (Figure 1). After 24 h incubation, rBbKl (50 µM) was able to reduce the viability of L929 cells by 50% (Figure 1A). Interestingly, inhibition of cell viability promoted by rBbKI was more effective after 48 h incubation, with an approximate inhibition of 80% (Figure 1B), which is quite promising. EcTI also demonstrated concentration-dependent inhibitory action, with approximately 80% inhibition occurring at a concentration of 50 µM after 72 h (Figure 1C). On the other hand, rBbCI did not significantly affect the viability of L929 cells under the analyzed conditions.

#### 2.2.2. Effects of Inhibitors on Cell Adhesion

**Figure 2:**
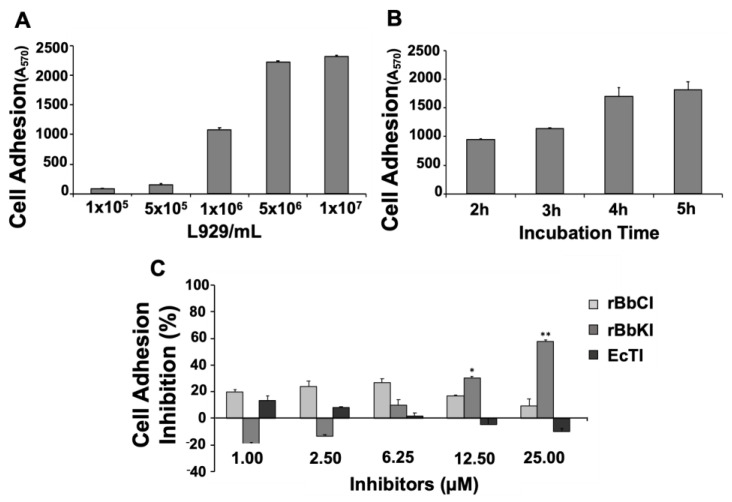
Action of rBbCI, rBbKI, and EcTI on cell adhesion of L929 cells. (**A**) Increasing concentrations of L929 were added to the 24-well plates previously coated with fibronectin and analyzed after 2 h of incubation. (**B**) 1 × 10^6^ L929 cells were added to 24-well plates previously coated with fibronectin and analyzed at different incubation periods (2, 3, 4, 5 h). (**C**) L929 cells and increasing concentrations of rBbCI, rBbKI, and EcTI were pre-incubated for 15 min at room temperature and added to 24-well plates previously coated with fibronectin. Each bar represents the mean ± standard deviations of three repetitions. (* *p* < 0.05, ** *p* < 0.005; One way-ANOVA, follow Tukey’s Multiple Comparison Test).

Cell adhesion is an essential process that can determine migration and generation of invasion process. Therefore, we analyzed the effect of the protease inhibitors on L929 cells coated with fibronectin, an important molecule that promote cell migration, invasion, and lung metastasis in soft-tissue sarcomas [30]. Two parameters were analyzed to establish ideal conditions for the adhesion assay with the fibrosarcoma cell line: the number of cells (1 × 10^5^, 5 × 10^5^, 1 × 10^6^, 5 × 10^6^, 1 × 10^7^ cell/mL) and the incubation time (2 h, 3 h, 4 h, and 5 h). The results indicated that the optimal response for the experimental procedures was obtained using 1 × 10^6^ cells in 2 h (Figure 2A,B).

We then investigated the effect of rBbCI, rBbKI, and EcTI (1.0–25 µM) on the adhesion of 1 × 10^6^ L929/mL to fibronectin after 2 h of incubation. The results demonstrated a dose-dependent inhibitory effect of rBbKI, which reached approximately 65% inhibition at a concentration of 25 µM, although a slight increase in cell attachment is noticed in the initial treatment (20%). On the other hand, rBbCI and EcTI did not significantly inhibit the adhesion of L929 to fibronectin under the conditions analyzed (Figure 2C).

#### 2.2.3. Effects of Inhibitors on the Cell Cycle

**Figure 3:**
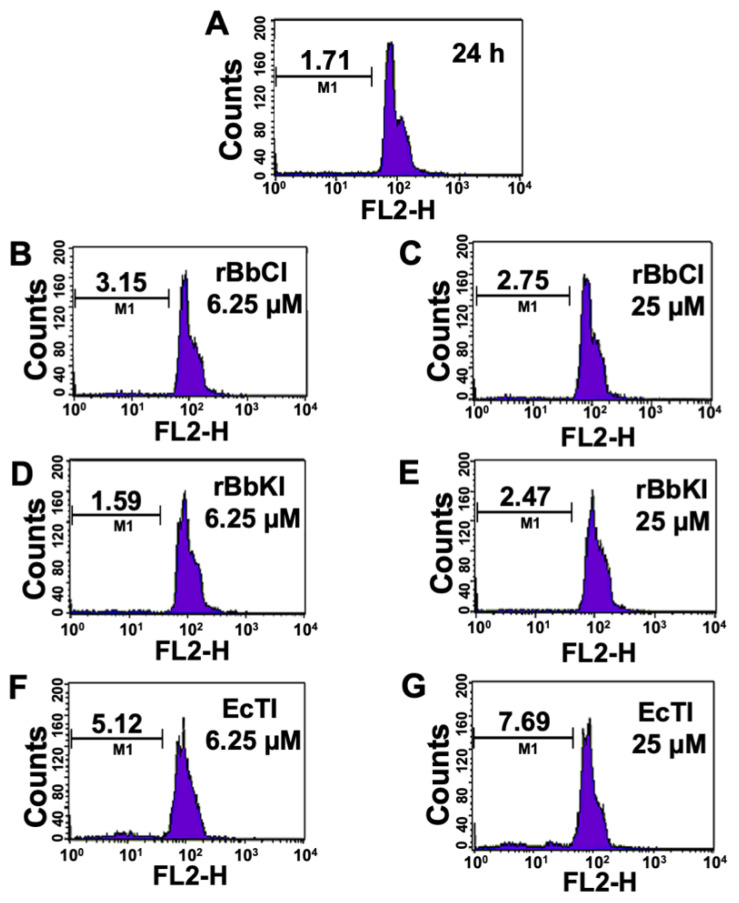
Effect of rBbCI, rBbKI, and EcTI on L929 cell cycle after 24 h incubation. L929 cells treated with (**A**) control, (**B**) 6.25 µM rBbCI, (**C**) 25 µM rBbCI, (**D**) 6.25 µM rBbKI, (**E**) 25 µM rBbKI, (**F**) 6.25 µM EcTI, and (**G**) 25 µM EcTI. M1 = fragmented cells.

Cancers have an exacerbated cell proliferation, controlled by the aberrant activity of important molecules of the cell cycle. Hence, cell cycle inhibitors are attractive for cancer therapies. For that, we investigated the effect of rBbCI, rBbKI, and EcTI (6.25 and 25 µM) on the cell cycle progression. The effects of the protease inhibitors were analyzed on the L929 cell at different incubation times (24 h—Figure 3, 48 h—Figure S1 and 72 h—Figure S2). None of the inhibitors provoked significant changes in the phases of the L929 cell cycle under the conditions analyzed.

#### 2.2.4. Confocal Microscopy

**Figure 4:**
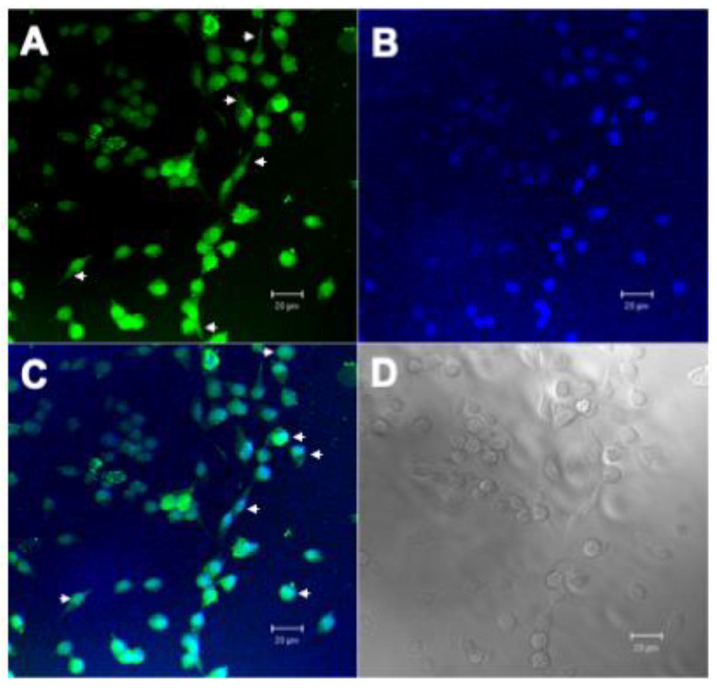
Confocal Microscopy Analysis of L929 with BbKI-AlexaFluor488. (**A**) L929 cells marked with BbKI conjugated with AlexaFluor 488 (green), showing cell surface localization (arrowheads), (**B**) L929 nuclei stained with 4′,6-Diamidino-2-phenylindole dihydrochloride, DAPI (blue), (**C**) overlay of images with BbKI nuclear localization (arrowheads) in L929 cells and (**D**) differential interference contrast (DIC) microscopy of L292 cells. Scale bar: 20 µm.

**Figure 5:**
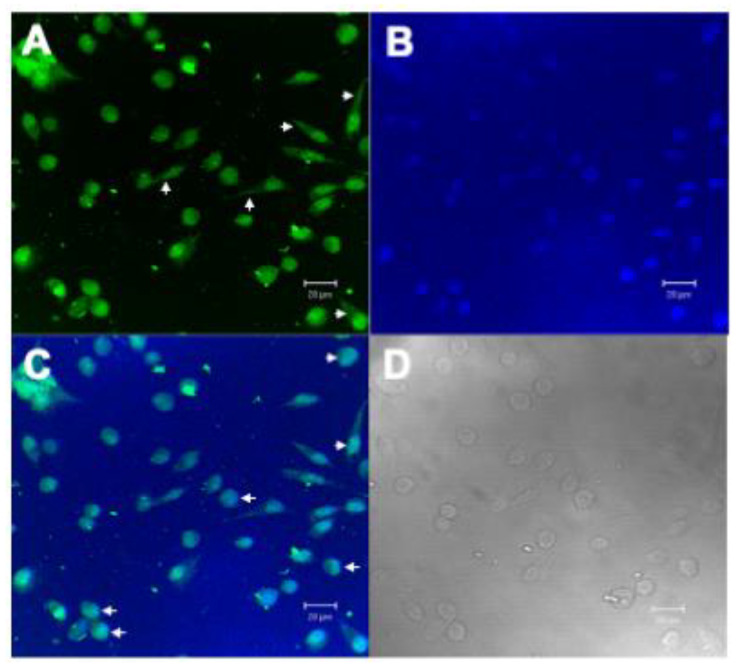
Confocal Microscopy Analysis of L929 with EcTI-AlexaFluor488. (**A**) L929 cells marked with EcTI conjugated with AlexaFluor 488 (green), showing cell surface localization (arrowheads), (**B**) L929 nuclei stained with 4′,6-Diamidino-2-phenylindole dihydrochloride, DAPI (blue), (**C**) overlay of images with EcTI nuclear localization (arrowheads) in L929 cells and (**D**) differential interference contrast (DIC) microscopy of L292 cells. Scale bar: 20 µm.

The main difficulty of compounds with therapeutic action is access to the inside of cancer cells. The plasma membrane is the first barrier formed by the cell against the entry of chemical compounds, making it difficult for antitumor molecules to access. Therefore, the search for molecular targets that have intracellular action is important. Once the inhibitory action of rBbKI and EcTI against L929 cells was observed in cell viability and cell adhesion, we analyzed the interaction of these proteins with the cell surface. For these experiments, proteins were labeled with AlexaFluor 488, which covalently binds to primary amines present in the protein structure and emits green fluorescence when excited with an argon laser at 488 nm (blue light), which is detected by fluorescence microscopy. Thus, L929 cells were incubated with previously labeled BbKI or EcTI (Figure 4A and Figure 5A).

The fluorescent dye DAPI was used to mark the location of the cell nucleus (Figure 4B and Figure 5B). Localization of the fluorescent-conjugated inhibitors indicated they bound to the cell surface and nucleus of L929 (Figure 4C and Figure 5C), which is relevant.

#### 2.2.5. Effect of the BbKI-Derived Peptide on L929 Cell Viability

**Figure 6:**
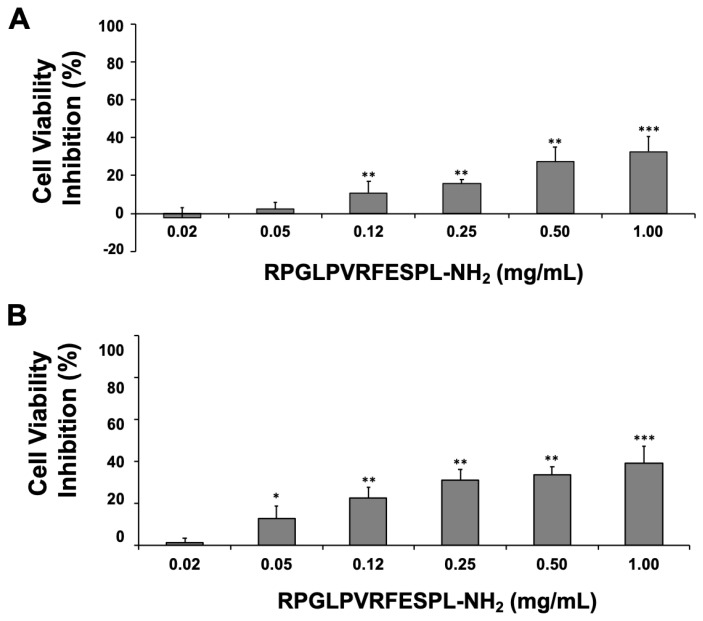
Action of the RPGLPVRFESPL-NH_2_ peptide on L929 cell viability after 24 and 48 h. L929 cells were pre-incubated for 24 h, increasing concentrations of the synthetic peptide were added to the 96-well plates, and cells were analyzed after incubation for (**A**) 24 h and (**B**) 48 h. Each bar represents the mean ± standard deviations of three repetitions. (* *p* <0.05, ** *p* <0.005, *** *p* <0.0001; One way-ANOVA, follow Tukey’s Multiple Comparison Test).

Employing the synthetic peptide RPGLPVRFESPL-NH_2_ derived from the BbKI reactive site to act on serine protease activity [31], we investigated the efficacy of BbKI peptide (0.02–1.0 mg/mL) on L929 cell viability at different incubation times (24 h—Figure 6A and 48 h—Figure 6B). The peptide inhibited cell viability in a dose-dependent manner, with maximum inhibition between 40% and 50%. These inhibitory levels did not change after 48 h, indicating the stability of the peptide.

## 3. Discussion

The pharmacological properties of protease inhibitors have been characterized and studied for decades in different biological models for therapeutic applications [8,9]. Their effectiveness may be due to direct inhibition of extracellular matrix (ECM) proteolysis or indirect inhibition of proteolytic cascade activation, thus preventing the spread of tumor cells, ECM degradation, and inhibition of tumor progression [8].

In the present study, we explored different inhibitory characteristics of plant protease inhibitors derivates: rBbCI, rBbKI, and EcTI by analyzing their efficacy against events elicited in the spread of tumors, such as proliferation, adhesion, and the cell cycle of mouse fibrosarcoma cells (L929). EcTI and BbKI are both serine protease inhibitors that contain arginine at the P1 position of the reactive site [10,26]; however, specificity differences demonstrated by these proteins made them interesting candidates for cellular studies, especially those of tumor origin. rBbKI and EcTI concentrations were inversely correlated with L929 cell viability, and no effect was detected with rBbCI treatment. Reduction in fibrosarcoma viability is interesting and relevant due to aggressivity and high recurrence rates of fibrosarcoma.

We determined that the action of EcTI was slower than that of rBbKI, inhibiting approximately 50% of cell viability after 24 h incubation, while EcTI reached a maximum of 20% inhibition in this period. However, after 48 h and especially 72 h, the EcTI inhibitory activity was high and similar (at 50 µM) to rBbKI. These differences probably reside in the inhibitory properties of each protease inhibitor. The action of EcTI and rBbKI must be important not only for inhibiting the activity of serine proteases, such as trypsin and chymotrypsin but also for inhibiting activation of a proteolytic cascade in which these enzymes are involved. In this case, the inhibitory activity of rBbKI against tissue kallikreins distinguishes it from EcTI. Tissue kallikreins are involved in cellular processes that lead to the activation of fundamental enzymes and receptors in the metabolism of these cells, such as protease-activated receptors [32,33]. BbKI can inhibit tissue kallikreins with K_iapp_ in the nM range, and this activity may be responsible for the faster inhibitory action of rBbKI compared with EcTI. Together with plasminogen activators, both BbKI and EcTI inhibit plasmin to form a powerful mechanism for generating proteolytic activity, which is necessary for tumor growth, metastasis, and angiogenesis. Plasminogen (the plasmin zymogen) plays an important role in metastasis as a primary tumor spreader [34,35]. Interestingly, BbCI, which is also present in the seeds of *B. bauhinioides* and exhibits 84% primary structure similarity with BbKI [36], demonstrates a distinguished inhibitory specificity by inhibiting cysteine protease cathepsin L and serine proteases elastase and cathepsin G [17]. Although increasing cathepsin L-like activity may be correlated with malignant tumors [37,38] and elastase [28], rBbKl did not affect L929 cell viability, proliferation, or cell adhesion. Thus, the inhibitory specificity of rBbKI may have impacted its toxicity towards fibrosarcoma cells.

Once rBbKI affected cell viability after 24 h and this effect was maintained after 48 h and 72 h, we investigated whether the peptide similar to the region of the reactive inhibitor site would be responsible for the effect of rBbKI on these cells. Although the greater potency of the protein compared with the peptide suggests other parts of the inhibitor structure may contribute complementary action, the difference may also be attributed to the change in specificity, as demonstrated by Cagliari et al. [31]. The peptide was not able to inhibit trypsin, and rBbKI is a potent inhibitor of this enzyme.

Degradation of extracellular matrix and adhesion is an essential process to the establishment of cancer cells. The role of fibronectin contributes to tumor malignancy, metastasis, and patients’ poor prognosis [30]. The protease inhibitor rBbKI was effective in reduce L929 cell adhesion to fibronectin, demonstrating the relevance of this protein in decreased cell viability and cell adhesion.

For a compound to have potentiated therapeutic action, it is necessary to access the cell via contact with proteins, lipids, or saccharides of the plasma membrane. In this view, molecules that have intracellular action are important. The hypothesis that the plant inhibitors could penetrate the cell was already demonstrated [20]. In the L929 line, the fluorescence microscopy showed rBbKI and EcTI interaction with the cell and nucleus surface, indicated by the green stain (Alexafluor 488), distributed along L929 cell and by blue stain (DAPI), dispersed by nuclear area demonstrating that rBbKI and EcTI were internalized. Further studies need to be conducted to investigate the consequences of cell signaling besides the effects on cell viability and adhesion.

Despite progress made with the use of these inhibitors, much remains unknown. Inhibitors may indirectly or directly signal or activate different components that induce cell death, providing prospects for studies to assess the importance of proteases involved in this process. The positive results obtained with the inhibitor fragment in this study indicate that the structures of these proteins warrant further exploration, such as cell signaling and in vivo experiments involved in this type of pathology.

## 4. Materials and Methods

### 4.1. Seeds

The seeds of *Bauhinia bauhinioides* (specimen number: 4665, CGMS 28770) and *Enterolobium contortisiliquum* (Vell.) Morong (specimen number: 10254, CGMS 56403) were collected in the region of Corumbá, Brazil, and identified by Dr. Vali Joana Pott (Brazilian Agricultural Research Corporation (Embrapa), Campo Grande, Brazil).

The species *B. bauhinioides* (Mart.) Macbr. belongs to the family Fabaceae. It is popularly known as cow’s hoff due to the shape of its leaves, which consist of two leaflets [35]. The species *E. contortisiliquum*, also belonging to the family Fabaceae, is a tree that grows over 20 m high, popularly known as ear pod due to its ear-shaped pods [25].

### 4.2. Purification of Native Inhibitors

The inhibitors from *B. bauhinioides* seeds (BbKI and BbCI) were purified according to the methodology described by Nakahata et al. [12]. The method described by de Paula et al. [11] was followed to obtain the EcTI inhibitor from *E. contortisiliquum* seeds. Some process modifications are detailed below.

### 4.3. Extraction of B. bauhinioides and E. contortisiliquum Inhibitors

**Figure 7:**
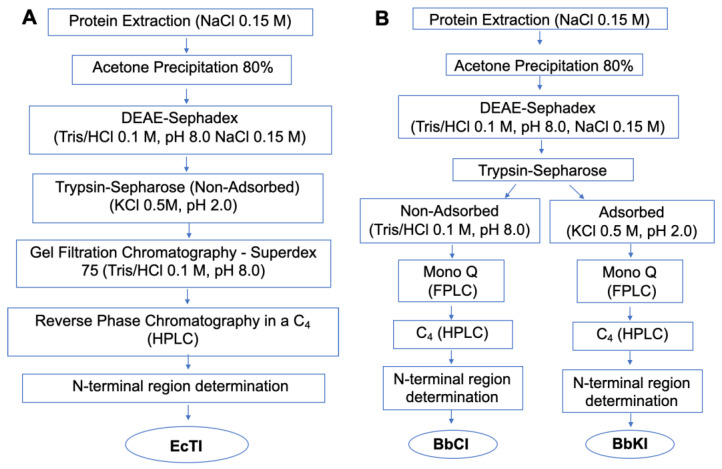

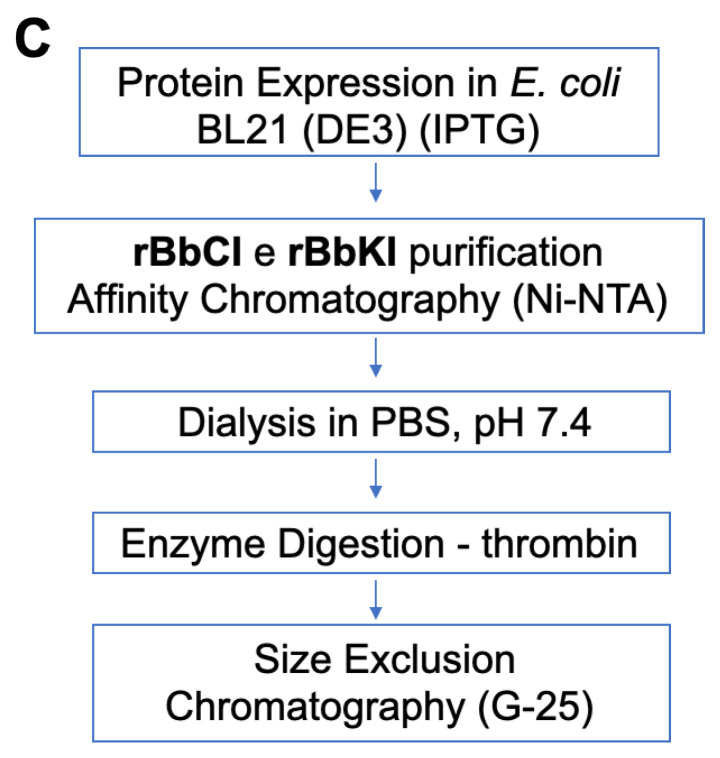
Purification scheme for (**A**) *Enterolobium contortisiliquum* trypsin inhibitor (EcTI), (**B**) *Bauhinia bauhinioides* cruzipain inhibitor (BbCI) and kallikrein inhibitor (BbKI), and (**C**) recombinant protein from BbCI (rBbCI) and BbKI (rBbKI).

Following the procedures shown in Figure 7A–C, *B. bauhinioides* and *E. contortisiliquum* seeds were crushed in a mill, and the resulting powder was homogenized at a 1:40 (*w*/*v*) ratio with 0.15 M NaCl in a blender. The material was centrifuged at 4000 rpm for 15 min at 4 °C and the supernatant was then heated to 56 °C for approximately 15 min before being slowly cooled in an ice bath. Proteins were precipitated with cold acetone, under slow and constant stirring at 4 °C, until reaching a final concentration of 80% (*v*/*v*). After the 30 min precipitation period, the material was centrifuged at 4000 rpm for 20 min at 4 °C. The protein precipitate was then vacuum-dried to evaporate residual acetone, solubilized in 0.15 M NaCl, followed by centrifugation under the same conditions described above.

### 4.4. Ion Exchange Chromatography on DEAE-Sephadex

The protein precipitate, after conductivity and pH adjustments, was applied to a DEAE-Sephadex A-50 column (3.0 × 13.0 cm, Amersham Biosciences, Piscataway, NJ, USA), previously equilibrated with 0.1 M Tris/HCl buffer (pH 8.0). The column was washed with equilibrium buffer until effluent Abs280 > 0.03. The bound protein was eluted with 0.1 M Tris/HCl buffer (pH 8.0) containing 0.15 M NaCl, followed by 0.1 M Tris/HCl buffer (pH 8.0) containing 0.3 M NaCl. Chromatography was performed under a constant flow of 30 mL/h, fractionated into 2 mL aliquots. Fractions with Abs280 > 0.05 were pooled. Pooled active fractions were dialyzed at 4 °C.

### 4.5. Affinity Chromatography on Trypsin-Sepharose

Dialyzed, pooled fractions containing inhibitory activity were chromatographed on a trypsin-Sepharose column (10.0 mL of resin) equilibrated in 0.1 M Tris/HCl buffer (pH 8.0). The column was washed with equilibration buffer until the effluent Abs280 > 0.03; non-bound material was collected. The column was subsequently washed with 0.1 M Tris/HCl buffer (pH 8.0) containing 0.15 M NaCl. The bound inhibitor was then eluted by acidification with 0.5 M KCl/HCl (pH 2.0) and the collected fractions (1.0 mL/min) were immediately neutralized by adding 1.0 M Tris/HCl solution (pH 9). Protein elution was followed by detecting Abs280, and inhibitory activity against trypsin (bound material) or HNE (human neutrophil elastase, non-bound material, in the case of BbCI) was followed by hydrolysis of Bz-Arg-pNan or MeO-Suc-Ala-Ala-Pro-Val-pNan substrates, as described below.

### 4.6. Fast Protein Liquid Chromatography on Mono Q

*B. bauhinioides* materials obtained from the trypsin-Sepharose affinity column (non-bound and bound material) were purified by fast protein liquid chromatography (FPLC) to remove possible contaminants. The Mono Q column (Amersham Biosciences, GE Healthcare, Amersham, UK) was equilibrated in 0.05 M Tris/HCl buffer (pH 8.0), and elution was performed using a gradient (0–0.5 M NaCl) with 0.05 M Tris/HCl buffer (pH 8.0) containing 0.5 M NaCl, at a flow rate of 0.5 mL/min. Fractions with inhibitory activity against HNE and trypsin, respectively, were pooled, and then lyophilized for purity analysis and enzyme inhibition studies.

### 4.7. Recombinant rBbCI and rBbKI Inhibitors

#### 4.7.1. Cloning

Cloning and purification of recombinant inhibitors rBbKI and rBbCI were conducted according to procedures described by Araújo et al. [39]. In summary, the genes encoding BbKI and BbCI were cloned by RT-PCR using degenerate primers, which were synthesized from protein sequences previously described by Oliva et al. [10] and de Oliveira et al. [17], respectively. The amplified product was inserted into the p-GEM T vector (Promega, Madison, WI, USA) for sequencing. Once sequences were confirmed, internal primers were synthesized to perform 5′ and 3′ RACE (Rapid Amplification of cDNA Ends). Both BbKI and BbCI were initially synthesized as pre-peptides with the following structure: 19 amino acid residues in the N-terminal region, 164 residues corresponding to the mature peptide, and 10 residues in the C-terminal region.

Sequences corresponding to the mature peptide were subcloned into the pET28a expression vector (Novagen, Madison, WI, USA). rBbKI and rBbCI inhibitors were produced by heterologous expression of a fusion protein consisting of a 6-residue histidine tail with a thrombin cleavage site between them. Transfected *E. coli* BL21 (DE3) (Merck KGaA, Darmstadt, Germany) produced inhibitors in large quantities, which were purified as described below.

#### 4.7.2. Expression of rBbCI and rBbKI Inhibitors

*E. coli* BL21 (DE3) colonies harbouring rBbKI and rBbCI were inoculated in 5 mL lysogeny broth (LB) with 50 μg/mL kanamycin, with agitation at 150 rpm for 12 h at 37 °C. The inoculum was then transferred to 500 mL LB containing 50 μg/mL kanamycin, agitated at 37 °C until Abs_600_ = 0.40–0.50. The culture was then induced for 3 h to express rBbCl and rBbKl inhibitors by adding 0.2 mM isopropyl β-d-1-thiogalactopyranoside (IPTG). At the end of the induction period, the material was centrifuged at 4000 rpm for 20 min at 4 °C, resuspended in 10 mL 0.1 M Tris/HCl buffer (pH 8.0) containing 0.15 M NaCl, and stored at −70 °C until use.

### 4.8. Purification of rBbKI and rBbCI Inhibitors

#### 4.8.1. Affinity Chromatography and Cleavage of the Fusion Peptide

Transfected *E. coli* were lysed by sonication (8 pulses of 30 s each) in an ice bath, followed by centrifugation at 10,000 rpm for 10 min at 4 °C. The supernatant was subjected to affinity chromatography using a Ni-NTA Superflow Column (2 mL resin; Qiagen, Hilden, Germany) previously equilibrated in 0.1 M Tris/HCl buffer (pH 8.0) containing 0.15 M NaCl. The column was washed with equilibration buffer and non-bound material was collected. Bound proteins were eluted with 0.1 M Tris/HCl buffer (pH 8.0) using an imidazole gradient (0.01 M, 0.1 M, 0.25 M, and 0.50 M). Elution of the rBbKI and rBbCI inhibitors was followed by detecting Abs_280_. Fractions eluted with 0.1 M Tris/HCl buffer (pH 8.0) containing 0.25 M and 0.50 M imidazole were dialyzed in sodium phosphate buffer (diluted PBS solution) at pH 7.4. Subsequently, samples were incubated with thrombin (0.5 U/mg protein) for 4 h at 18 °C and immediately subjected to size-exclusion chromatography.

#### 4.8.2. Size Exclusion Chromatography

Fractions containing native inhibitors obtained from trypsin-Sepharose chromatography, as well recombinant forms obtained by thrombin cleavage, were purified by size exclusion chromatography using the Äkta system (GE Healthcare, Chicago, IL, USA). A Superdex 75 10/300 GL column (Amersham Biosciences, GE Healthcare, Amersham, UK) was equilibrated with 0.1 M Tris/HCl buffer (pH 8.0), and previously calibrated with different molecular mass proteins (ovalbumin, 45 kDa; carbonic anhydrase, 30 kDa; soybean trypsin inhibitor, 20 kDa; cytochrome C, 12.4 kDa). Chromatography was performed under a constant flow of 0.5 mL/min, fractionated in 1 mL aliquots. Inhibitor elution was followed by detecting absorbance at 280 nm.

#### 4.8.3. Reverse-Phase Chromatography (HPLC System)

Fractions containing inhibitory activity were concentrated by lyophilization and purified by reverse-phase chromatography (C4 column 15 × 0.5 cm; Beckman Ultrasphere, Lake Forest, CA, USA) equilibrated in 0.1% trifluoroacetic acid (TFA) in water. Elution was performed using an acetonitrile gradient (90% (*v*/*v*), 0–100% in 0.1% TFA in water), under a constant flow of 0.7 mL/min. Sample homogeneity was confirmed by SDS-PAGE (data not shown).

#### 4.8.4. Enzymatic Assays

Enzymes, substrates and specific buffers were used to assess substrate hydrolysis by the proteases (Table 2). The substrates used in each experiment demonstrated optimum specificity for the enzyme tested [12]. Chromogenic substrates derived from p-nitroanilide (Calbiochem, Darmstadt, Germany) were employed for highly sensitive photometric detection of p-nitroaniline released after enzymatic hydrolysis, using a SpectraCount spectrophotometer (Hewlett-Packard, Palo Alto, CA, USA) at 405 nm. Substrates were initially diluted in dimethyl sulfoxide (DMSO) and further dilutions were performed in an appropriate buffer for each assay. Fluorogenic substrates (Calbiochem) derived from 7-amino-4-methylcoumarin (AMC) were diluted in dimethylformamide (DMF); hydrolysis was monitored at 380 nm (excitation) and 460 nm (emission) using a FluoroCount spectrofluorometer (Hewlett-Packard). Assays using substrate Abz-X-EDDnp (diluted 1:1 (*v*/*v*) in DMF and water) were performed in an F-2000 spectrofluorometer (Hitachi, Tokyo, Japan) with hydrolysis detection at 320 nm (excitation) and 420 nm (emission).

Apparent inhibition constants were determined by calculating the dissociation constant values of the enzyme-inhibitor complex (K_iapp_), following the model proposed by Morrison (1989) [40]. Enzymatic kinetics were calculated using the GraFit© Version 3.0 (Erithacus Software Ltd., Horley, UK).

#### 4.8.5. Hydrolysis of Substrates by Serine Proteases and Determination of Inhibitory Activity

Serine protease activity was assessed on specific substrates, using enzymes that were pre-incubated at 37 °C with different inhibitor concentrations in an appropriate buffer. The substrate was added (250 µL final volume) after 10 min (Table 2), incubated for 20–30 min (depending on the enzyme) at 37 °C, and the reaction was interrupted with 40 µL 40% acetic acid. Substrate hydrolysis was monitored by detecting Abs_405_ from released p-nitroaniline, or fluorescence at 380 nm (excitation) and 460 nm (emission wavelengths) from AMC release. Inhibitory activity was calculated by determining the residual enzyme activity in the assays. Inhibitor concentrations were calculated assuming 1:1 reaction stoichiometry. This methodology was also used to identify inhibitory activity during the purification processes. Experiments were performed in triplicate.

### 4.9. Hydrolysis of Z-Phe-Arg-AMC by Cysteine Proteases and Determination of Inhibitory Activity

Cysteine protease concentrations were obtained through titrations with egg cystatin [41]. Inhibition of cathepsin L, cruzipain, or cruzain was determined through residual enzyme activity on the substrate Z-Phe-Arg-AMC. Enzymes were activated by incubation for 10 min at 37 °C in 0.1 M Na_2_HPO_4_ buffer (pH 6.3) containing 10 mM EDTA, 400 mM NaCl, and 2 mM DTT. In typical experiments performed with the same activation buffer, cathepsin L (18 nM), cruzipain (18 nM), or cruzain (3.2 nM) was pre-incubated with increasing concentrations of purified inhibitor for 10 min at 37 °C, and then the substrate Z-Phe-Arg-AMC (0.3 mM) was added. Substrate hydrolysis was monitored by detecting fluorescence at 380 nm (excitation) and 460 nm (emission) wavelengths, using an F-2000 spectrofluorometer (Hitachi). The increase in fluorescence was continuously recorded for 10 min. Experiments were performed in triplicate. Residual activity was determined by comparing enzymatic hydrolysis curves in the presence and absence of the inhibitor.

#### 4.9.1. Determination of rBbKI and EcTI Concentrations

Increasing amounts of rBbKI and EcTI were pre-incubated for 10 min at 37 °C in 0.05 M Tris/HCI buffer (pH 8.0) containing 0.02% (*v*/*v*) CaCl_2_ with trypsin previously titrated with 4-guanidinobenzoic acid 4-nitrophenylester hydrochloride (NPGB). The substrate Bz-Arg-pNan (25 uL, 10.0 mM) was then added to the pre-incubated sample (250 mL final volume). Substrate hydrolysis was monitored by detecting Abs405 of the released p-nitroaniline. Inhibitor concentrations were calculated assuming 1:1 reaction stoichiometry [10].

#### 4.9.2. Determination of rBbCI Concentration

Increasing amounts of rBbCI were pre-incubated for 10 min at 37 °C in 0.1 M Tris/HCI buffer (pH 7.0) containing 0.5 M NaCl and 17 nM HNE. Substrate MeO-Suc-Ala-Ala-Pro-Val-pNan (25 µL, 11.0 mM) was then added to the pre-incubated sample (250 mL final volume). Substrate hydrolysis was monitored by detecting Abs_405_ of the released p-nitroaniline. Inhibitor concentrations were calculated assuming 1:1 reaction stoichiometry.

#### 4.9.3. Synthetic Peptide RPGLPVRFESPL-NH_2_

The peptide Arg-Pro-Gly-Leu-Pro-Val-Arg-Phe-Glu-Ser-Pro-Leu-NH_2_ (RPGLPVRFESPL-NH_2_), derived from the reactive site of BbKI, was previously described by Oliva et al. [10] and Cagliari et al. [31]. The peptide was synthesized at the Departamento de Biofísica, UNIFESP (São Paulo, Brazil).

#### 4.9.4. Cultivation Conditions for the L929 Cell Line

The mouse fibrosarcoma strain L929 was maintained in RPMI medium (pH 7.4) supplemented with 10% fetal bovine serum (FBS; Invitrogen, Carlsbad, CA, USA), penicillin (100 U/mL), and streptomycin (100 µg/mL) at 37 °C under 5% CO_2_ until cells reached 80–90% confluence, as described below.

For the maintenance of cell stocks, the medium was initially removed from the culture of confluent cells, which were subsequently washed once with PBS buffer (pH 7.4) containing 140 mM NaCl, 1.7 mM KH_2_PO_4_, and 2.7 mM KCl. Cells were detached using a 2.5% trypsin solution for approximately 1 min. Cells were then carefully resuspended several times using a serological pipette and transferred to a tube, followed by centrifugation at 2000 rpm for 3 min, at 25 °C. Finally, cells were resuspended in RPMI medium (pH 7.4) with 10% FBS, and 5 × 10^4^ cells were transferred to a new bottle containing RPMI medium (pH 7.4) supplemented with 10% FBS. The culture medium was renewed every 2–3 days. Cells were used for experiments when they reached approximately 80–90% confluence.

#### 4.9.5. Cell Viability

L929 cells were washed with sterile PBS buffer (pH 7.4), removed from the culture bottle with trypsin solution (2.5%, *v*/*v*), centrifuged, and suspended in RPMI (pH 7.4) with 10% FBS. Cells were then counted in a Neubauer chamber. Cells (1 × 10^4^ cells/100 µL/well) were incubated at 37 °C under 5% CO_2_ for 24 h in RPMI medium (pH 7.4) with 10% FBS to allow adhesion.

rBbCI, rBbKI, and EcTI inhibitors at concentrations ranging from 1.0–50 µM were diluted in previously filtered (0.22 µm; Millipore, Billerica, MA, USA) RPMI medium (pH 7.4) with 10% FBS. Inhibitors were added to the L929 cell-containing wells and incubated at 37 °C under 5% CO_2_ for 24, 48, and 72 h. The synthetic peptide (RPGLPVRFESPL-NH_2_) was diluted first in DMSO (final concentration 1% DMSO) and then diluted in RPMI medium (pH 7.4) with 10% FBS at concentrations ranging from 0.02–1.0 mg/mL. Synthetic peptide was added to the wells, and incubated at 37 °C under 5% CO_2_ for 24 and 48 h. At the end of each incubation period, 10 µL MTT (5 mg/mL) was added to each well, and cells were incubated for 2 h at 37 °C under 5% CO_2_. The culture medium was then removed, and isopropanol was added for an additional 20 min at 37 °C. Finally, Abs_620_ was measured using a SpectraCount spectrophotometer (Hewlett-Packard). Assays were performed in triplicate for each concentration of inhibitor and peptide.

#### 4.9.6. Cell Adhesion Assay

Cell adhesion assays were performed according to the method described by de Paula et al. [11], with some modifications. Briefly, 24-well plates were pre-coated with 10 µg/250 µL/well fibronectin diluted in PBS (pH 7.4), and incubated at 37 °C under 5% CO_2_ for 2 h or overnight at 4 °C. The protein-binding site not yet covered by fibronectin was blocked by incubation with 250 µL/well 1% BSA in sterile PBS (pH 7.4) for 1 h at 37 °C. After excess BSA was removed, the plates were washed 3× with PBS (pH 7.4).

L929 cells were removed from the culture bottle using trypsin solution (2.5% *v*/*v*), followed by washing with sterile PBS (pH 7.4). Cells were then centrifuged at 2000 rpm for 3 min, at 25 °C and suspended in RPMI (pH 7.4). Cells (1 × 10^6^ cells/100 µL/well) and 1.0–25 µM inhibitor (200 µL/well) diluted in sterile RPMI (pH 7.4) were pre-incubated for 15 min at room temperature, added to the cell adhesion plates, and incubated at 37 °C under 5% CO_2_ for 2 h. At the end of the incubation period, non-adherent cells were removed by washing 3× with PBS (pH 7.4). Methanol was added to the wells and incubated for 5 min at 25 ºC to fix the adhered cells. Subsequently, the plates were washed 3× with PBS (pH 7.4) and stained with 1% toluidine blue solution in 1% borax for 5 min. Wells were washed 4× with PBS (pH 7.4), and pigment retained by cells was released with a 1% SDS solution. Absorbance at 570 nm (A570) was measured using a SpectraCount spectrophotometer (Hewlett-Packard). Assays were performed in triplicate for each concentration of inhibitor.

#### 4.9.7. Cell Cycle Assay

L929 cells (1 × 10^4^ cells/mL) were diluted in RPMI (pH 7.4) and cultured in 60 × 10 mm plates for 6 h at 37 °C under 5% CO_2_. The medium was then removed and cells were maintained in RPMI medium (pH 7.4) supplemented with 10% FBS for 24 h. rBbCI, rBbKI, and EcTI inhibitors were added at concentrations of 6.25 and 25 µM, followed by incubation for 24, 48, and 72 h at 37 °C under 5% CO_2_. After washing with PBS (pH 7.4), the medium and adhered cells were removed and pooled. Samples were centrifuged at 2000 rpm for 4 min at 25 °C, the supernatant was discarded, 5 mL PBS (pH 7.4) was added to the precipitate, and the sample was again centrifuged. Cells were then resuspended in 0.5 mL PBS (pH 7.4), and 0.5 mL absolute ethanol was added slowly under constant agitation. This material was stored at 4 ° C until use when it was again centrifuged, and the ethanol was carefully removed. The cells were then washed with 1 mL PBS (pH 7.4), and centrifuged at 2000 rpm for 4 min. The cells were resuspended in 100 µL PBS (pH 7.4) and 400 µL of propidium iodide (50 µg/mL) [25]. Cells were incubated for 30 min at 4 °C in the absence of light and immediately analyzed using a flow cytometer (FACS; Becton-Dickinson, San Diego, CA, USA).

### 4.10. Analysis of Cell Interaction with Inhibitors by Confocal Microscopy

#### 4.10.1. Covalent Conjugation of Inhibitors to Fluorescent Dye

EcTI and BbKI inhibitors (1 mg/mL) were solubilized in 200 µL 0.1 M sodium bicarbonate (pH 9.0). AlexaFluor 488 (1 mg/mL) was solubilized in 100 µL DMSO. Incubation of each inhibitor with AlexaFluor 488 (1:15 protein/fluorophore molar ratio) was carried out in a nitrogen atmosphere at 25 °C in the absence of light, and under constant agitation for 3 h. After incubation, the solution containing the protein and fluorophore was subjected to size exclusion chromatography on a G-25 gel against 0.03 M HEPES buffer (pH 7.4) to eliminate excess fluorophore [11].

#### 4.10.2. Cell Labeling for Confocal Microscopy

Glass coverslips (10 mm) in a 24-well plate were covered with 0.1 mg/mL poly-D-lysine in sterile PBS (pH 7.4), and maintained at 37 °C for 2 h. After this period, excess poly D-lysine solution was removed and the coverslips were dried at room temperature under laminar flow for 4 h [19].

Subsequently, 1 mL RPMI medium (pH 7.4) supplemented with 10% FBS containing 1 × 10^5^ L929 cells, was placed carefully on each coverslip and cultured at 37 °C for 24 h. The cells were then washed with PBS (pH 7.4) at 4 °C and fixed with 2% paraformaldehyde (*v*/*v*) solution. After 30 min, the cells were washed with PBS (pH 7.4) containing 0.1 M glycine, and incubated for 1 h with 40 µg/mL AlexaFluor 488-conjugated inhibitors. Then, the cells were incubated for 15 min with 25 µg/mL DAPI diluted 1:400 with PBS (pH 7.4) plus 0.01% (*w*/*v*) saponin. Then, the coverslips were washed with PBS (pH 7.4) and fixed with 5 µL Fluoromont-G for analysis by confocal microscopy. The solutions were kept in an ice bath throughout the cell labeling process. Analysis of the slides was performed using a Zeiss Axiophot fluorescence microscope and a LSM 510 laser scanning confocal microscope (Zeiss, Wetzlar, Germany).

### 4.11. Statistical Analysis

Statistical significance was determined by One-Way ANOVA followed by Tukey’s post-test to compare the means between independent groups. All experiments were performed in triplicate. Reproducible results were obtained, and representative data are shown. The values of * *p* ≤ 0.05, ** *p* ≤ 0.005 or *** *p* ≤ 0.0005 were accepted as significant.

## Figures and Tables

**Table 1:** Inhibitory properties of BbCI, rBbCI, BbKI, rBbKI, and EcTI (K_iapp_ values are shown in nM).

Enzyme	BbCI	rBbCI	BbKI	rBbKI	EcTI
Trypsin	φ	φ	20.00	28.00	0.88
Chymotrypsin	φ	φ	26.00	ND	1.11
Plasmin	φ	φ	330.00	ND	9.36
HNE	5.30	1.70	φ	φ	55.0
Factor Xa	φ	ND	φ	ND	φ
Thrombin	φ	ND	φ	ND	ND
PKa	φ	φ	2.40	2.00	6.15
PoPK	φ	φ	200.00	900.00	φ
Cathepsin G	160.00	ND	φ	φ	ND
Cathepsin L	0.22	ND	φ	ND	ND
Cruzain	0.30	0.30	φ	φ	ND
Cruzipain	1.30	1.20	φ	φ	ND

HNE: human neutrophil elastase; PKa: human plasma kallikrein; PoPK: porcine pancreatic kallikrein, φ: indicates a lack of measurable inhibition; ND: not determined.

**Table 2:** Enzymes, substrates and buffer used to verify substrate hydrolysis by serino proteases and inhibitory activity.

Enzyme	Substrate	Buffer
trypsin (20 μL, 0.41 μM) * NPGB	Bz-Arg-pNan (25 μL, 10 mM)	Tris/HCl 0.1 M, pH 8.0, CaCl_2_ 0.02% (*v*/*v*)
HNE (20 μL, 0.21 μM) (* α_1_-anti-trypsin)	MeO-Suc-Ala-Ala-Pro-Val-pNan (25 μL, 1.1 mM)	Tris/HCl 0.1 M, pH 7.0, NaCl 0.5 M
PKa (20 μL, 0.84 μM) (* EcTI)	H-D-Pro-Phe-Arg-pNan (25 μL, 5 mM)	Tris/HCl 0.05 M, pH 8.0
PoPK (30 μL, 0.16 nM) (* aprotinin)	H-D-Pro-Phe-Arg-AMC (30 μL, 5 mM)	Tris/HCl 0.1 M, pH 9.0, plus albumin 0.1% (*v*/*v*)
chymotrypsin (40 μL, 0.88 µM) (* EcTI)	Suc-Phe-pNan (20 μL, 20 mM)	Tris/HCl 0.1 M, pH 8.0, CaCl_2_ 0.02% (*v*/*v*)
plasmin (25 μL, 0.028 μM) (* BvTI)	H-D-Val-Leu-Lys-pNan (20 μL, 9 mM)	Tris/HCl 0.1 M, pH 7.4, NaCl 0.2 M
thrombin (10 μL, 0.267 μM) (* rhodinin)	H-D-Phe-L-Pip-L-Arg-pNan (20 μL, 2 mM)	Tris/HCl 0.05 M, pH 8.0
factor Xa (30 μL, 0.467 μM) (* BuXI)	Boc-Ile-Glu-Gly-Arg-AMC (60 μL, 6 mM)	Tris/HCl 0.05 M, pH 8.0
cathepsin G (30 μL, 0.25 μM) (* α_1_-anti-trypsin)	N-Suc-Ala-Ala-Pro-Phe-pNan (25 μL, 1 mM)	Tris/HCl 0.05 M, pH 7.0, NaCl 0.5 M

* inhibitor used for enzyme titration. HNE: human neutrophil elastase; PKa: human plasma kallikrein; PoPK: porcine pancreatic kallikrein.

## Data Availability

There is no data.

## Supplementary Materials

The following are available online at https://www.mdpi.com/2223-7747/10/3/602/s1, Figure S1: Effect of rBbCI, rBbKI, and EcTI on L929 cell cycle after 48 h incubation, and Figure S2: Effect of rBbCI, rBbKI, and EcTI on L929 cell cycle after 72 h incubation.
