# Supplementary material for: Differences in the Inhibitory Specificity Distinguish the Efficacy of Plant Protease Inhibitors on Mouse Fibrosarcoma
Source: Plants (Basel). 2021 Mar 23;10(3):602. doi: 10.3390/plants10030602 (PMC8005126; doi:10.3390/plants10030602)
Supplement: Supplementary file 1 [file plants-10-00602-s001.pdf]

*Supplementary Figure*

# Differences in the Inhibitory Specificity Distinguish the Efficacy of Plant Protease Inhibitors on Mouse Fibrosarcoma

**Sonia Yoo Im [1], Camila Ramalho Bonturi [1¥], Adriana Miti Nakahata [2], Clóvis Ryuichi Nakaie [3], Arnildo Pott [4], Vali Joana Pott [4] and Maria Luiza Vilela Oliva [1,*]**

**¥ These authors contributed equally to the present work.**

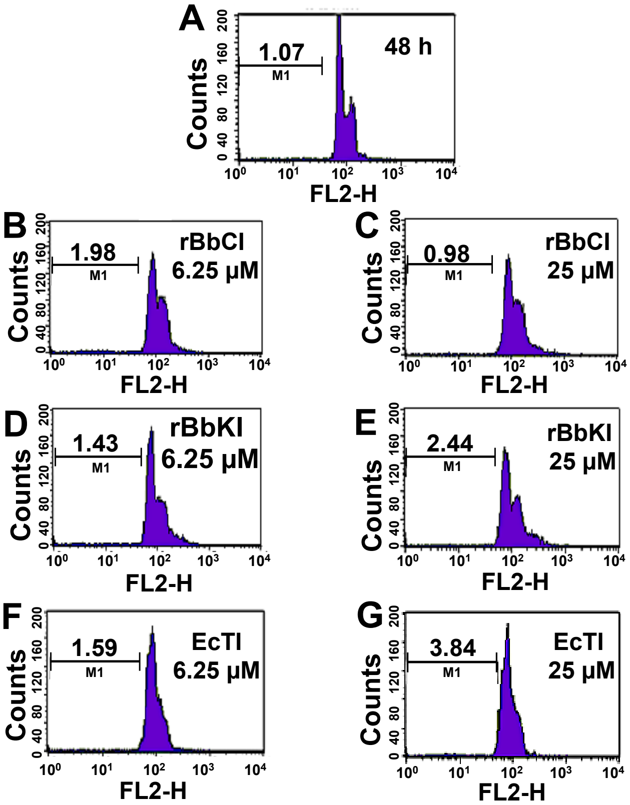


**Figure S1.** Effect of rBbCI, rBbKI, and EcTI on L929 cell cycle after 48 h incubation. L929 cells treated with (**A**) control, (**B**) 6.25 µM rBbCI, (**C**) 25 µM rBbCI, (**D**) 6.25 µM rBbKI, (**E**) 25 µM rBbKI, (**F**) 6.25 µM EcTI, and (**G**) 25 µM EcTI. M1 = fragmented cells.

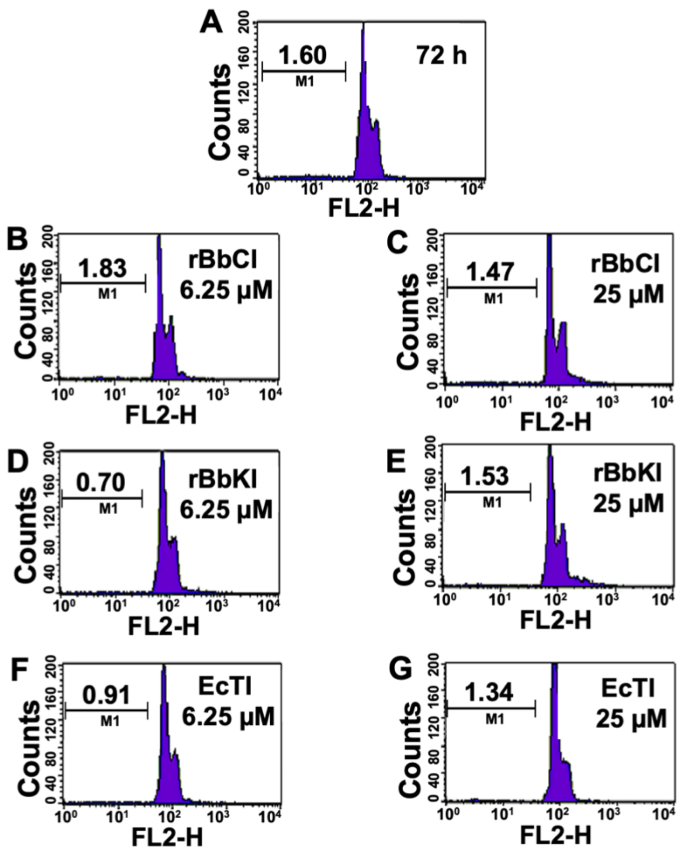


**Figure S2.** Effect of rBbCI, rBbKI, and EcTI on L929 cell cycle after 72 h incubation. L929 cells treated with (A) control, (B) 6.25 µM rBbCI, (C) 25 µM rBbCI, (D) 6.25 µM rBbKI, (E) 25 µM rBbKI, (F) 6.25 µM EcTI, and (G) 25 µM EcTI. M1 = fragmented cells.
